# Colorimetric changes of zirconia-reinforced lithium disilicate with varied thicknesses under UV aging conditions

**DOI:** 10.34172/joddd.41390

**Published:** 2024-12-14

**Authors:** AbdelRahman Moammad Abdelhameed, Hussein Ramadam Mohammed, Ahmad Mohammad Yousri El Kouedi

**Affiliations:** Crown and Bridge Department, Faculty of Dental Medicine, Al Azhar University, Cairo, Egypt

**Keywords:** Accelerated artificial aging, Colorimetry, Dental porcelain, Glass ceramics, IPS e.max press, Translucency, Vita Ambria, Zirconia-reinforced lithium silicate

## Abstract

**Background.:**

Zirconia-reinforced lithium disilicate (ZLD) is a promising material for esthetic dental restorations due to its strength, translucency, and color stability. However, its durability under accelerated aging conditions needs further investigation. The present in vitro study evaluated the effect of UV accelerated aging on the translucency and color stability of ZLD at varying thicknesses, using IPS e.max Press (LD) as a reference.

**Methods.:**

Seventy-two samples were divided into two groups: high translucent (HT) Vita Ambria (ZLD) and IPS e.max Press (LD) (n=36, each). Each group was further subdivided into four thicknesses: 0.5, 1.0, 1.5, and 2.0 mm (n=9). The samples were fabricated, polished, and subjected to UV weathering for 384 hours, simulating one year of clinical service. Translucency and color changes were assessed using a spectrophotometer. Data were analyzed using SPSS 20, with independent t-test, paired t-test, and one-way ANOVA at a significance level of *P*≤0.05.

**Results.:**

Vita Ambria exhibited significantly higher translucency before and after aging compared to IPS e.max press at all thicknesses (*P*=0.000). In both materials, translucency decreased when the thickness increased (*P*=0.000), observed before and after UV aging. Vita Ambria also displayed a greater color change (ΔE=2000) compared to IPS e.max press across all thicknesses (*P*=0.000).

**Conclusion.:**

ZLD exhibited higher translucency than LD before and after accelerated artificial aging, indicating that the accelerated aging process adversely impacted the optical properties of the tested material. However, LD demonstrated superior color stability.

## Introduction

 Glass ceramics have become increasingly popular in dental practice due to their ability to meet esthetic demands.^1–3^ This class of ceramic materials possesses a unique combination of properties, including translucency, color stability, and biocompatibility, making them highly suitable for use in fixed dental prosthodontics.^4,5^ Through careful adjustments in composition and manufacturing techniques, glass ceramics have been tailored to closely replicate the optical properties of natural enamel.^1,4^

 Similarly, lithium disilicate-based glass ceramics have been firmly established for esthetic purposes.^1,6,7^ Its remarkable combination of strength and natural-looking translucency makes it an ideal choice for creating dental restorations that seamlessly blend with the patient’s existing teeth. Whether crowns, bridges, or veneers, lithium disilicate delivers both functional durability and esthetic appeal, enhancing smile and restoring function.^8-13^

 Translucency and color stability play a crucial role in the esthetic application of dental materials. The ability of a material to maintain its color over time, especially under the challenging conditions of the oral environment, is important for the longevity of dental restorations.^14-16^

 The International Commission on Illumination (CIE) has devised a color system that evaluates the chromaticity of dental materials in a consistent three-dimensional model. Discoloration can be assessed by examining differences between the Lab* color coordinates. The CIEDE2000 color difference formula (∆E) is recommended for in vivo color analysis and dental research because it corrects the non-uniformity found in the CIELab color space, particularly for minor variations in color.^17^ According to a study by Ghinea et al., the CIEDE2000 formula provides a better fit than the CIELab formula when measuring color differences and determining acceptability and perceptibility in dental ceramics.^18-20^

 Translucency, on the other hand, is essential for the natural appearance of dental restorations.^21,22^ This property determines the material’s ability to transmit light, directly influencing its visual appearance and capacity to blend seamlessly with natural dentition. Achieving an optimal level of translucency is essential in dentistry, as it enables restorations to replicate the natural light dynamics of surrounding teeth, thereby giving them a lifelike vitality.^23-26^

 There is a growing demand for dental materials that enhance the appearance of teeth. Zirconia-reinforced lithium disilicate (ZLD) has emerged as a potential alternative to lithium disilicate for esthetic purposes.^27,28^ Adding zirconia particles in lithium-based ceramics was found to improve the strength and the optics of the material.^1,4,10^ Currently, the commercial ZLD ceramic system, Vita Ambria (VITA Zahnfabrik, Bad Sackingen, Germany), is offered as pressable ingots, which features a unique microstructure. This microstructure includes tetragonal zirconia fillers (ZrO_2_) at a concentration of 8%‒12% within its glassy matrix, serving as a secondary nucleating agent.^4,29,30^

 The available studies on Vita Ambria are limited, but most have focused on evaluating various mechanical and biological aspects of the material, including margin adaptation,^9,10,27^ bond strength,^31^ flexural strength,^32^ cantilever design,^33^ internal fit,^9,34^ and fracture resistance.^9^ However, fewer studies have examined the optical properties of the material. Rizk et al^14^ compared Vita Ambria, IPS e.max Press, and other materials to assess the impact of accelerated aging on their translucency. However, the study limited its scope to a thickness of 1.5 mm, and the accelerated aging method employed was thermocycling.

 Artificial accelerated aging (AAA) methods are techniques used to evaluate the durability of glass ceramic materials in prosthodontic applications.^15,17^ Various techniques, such as hydrothermal exposure, thermocycling, and thermomechanical processes, are employed for this purpose. Each technique simulates specific oral conditions and loading scenarios, offering valuable insights into material behavior.^10,14,15^

 Ultraviolet (UV) weathering is a vital technique in artificial aging studies that exposes materials to contr,.olled UV radiation doses to replicate prolonged sunlight exposure.^35^ Thismethod induces surface degradation, aiding researchers in assessing material stability and longevity.^15,36,37^ In light of these considerations, this in vitro study explored the effects of UV accelerated aging on the translucency and color stability of ZLD with varying thicknesses. Therefore, the null hypothesis of this study was that there would be no significant difference in the mean translucency parameter (TP) and color difference (ΔE2000) between Vita Ambria and IPS e.max Press glass ceramics with varying thicknesses after UV-accelerated aging.

## Methods

 All the materials used in this study and their technical information are listed in Table 1.

**Table 1:** Technical information of the materials used in the clinical study

**Material**	**Description**	**Composition**	**Manufacturer**	**Translucency and shade**
Vita Ambria	Zirconia-reinforced lithium disilicate glass-ceramic	SiO_2_ 58-66%, Li_2_O 12-16%, ZrO_2_ 8-12%, Al_2_O_3_1-4%, P_2_O_5_ 2-6%, K_2_O 1-4%, B_2_O_3_ 1-4%, CeO_2_ 0-4%, Tb_4_O_7_ 1-4%, V_2_O_5_ < 1%, Er_2_O_3_ < 1%, Pr_6_O_11_ < 1%	VITA Zahnfabrik, Bad Sackingen, Germany	HT/A2
IPS e.max press	Lithium disilicate glass-ceramic	SiO_2_ 58-80%, Li_2_O 11-19%, K_2_O 0-13%, ZnO_2_ 0-8%, Al_2_O_3_ 0-5%, P_2_O_5_, MgO, and other oxides.	Ivoclar Vivadent AG, Schaan, Liechtenstein	HT/A2

###  Ceramic sample fabrication

**Figure 1:**
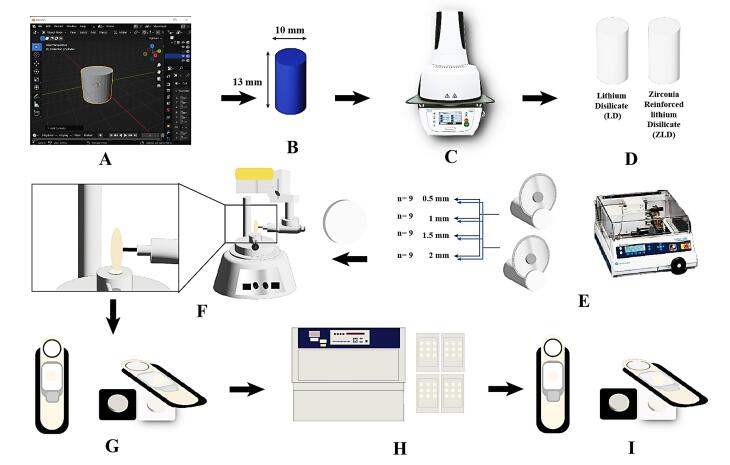
A diagram illustrating the experimental steps in this study. A, designing the 3D cylinder using Blender 3D software. B, milled wax cylinders. C, pressing step using the Ivoclar Vivadent Programat EP 3010 pressing furnace. D, ceramic cylinders obtained from LD and ZLD. E, IsoMet used to cut the ceramic discs into different thicknesses. F, polishing the ceramic samples using a milling surveyor. G, baseline translucency and color measurements with the spectrophotometer. H, artificial aging using the Q-lab weatherometer. I, final color measurements

 Based on Babaier et al^23^ and using the G*Power statistical power analysis program (SPSS Chicago, IL, USA, version 3.1.9.4) for sample size determination, a total sample size of 72 was deemed sufficient. The samples were divided according to ceramic material (36 each) and further subdivided into four subgroups (n = 9) according to different thicknesses (Figure 1E).

 A schematic flowchart of the experimental steps is summarized in Figure 1. All the ceramic samples, with a uniform diameter of 10 mm but varying thicknesses, were fabricated using the following technique. Initially, A 3D cylinder (10 mm in diameter and 13 mm in length) was designed using Blender V2.80 software (Blender - a 3D modeling and rendering package; Stichting Blender Foundation, Amsterdam). Following the design phase, precision milling from wax was conducted using the Sirona inLab MC X5 Dental Milling Machine InLab MC X5 (Sirona Dental Systems, GmbH, Bensheim, Germany) (Figure 1B).

 Subsequently, 10 wax cylinders were used to produce 10 ceramic cylinders of identical dimensions. This was achieved by applying the heat-pressing technique (Figure 1C, D). The 10 ceramic cylinders (5 from each material) were inspected for cracks, voids, or defects. If any flaws were found, the cylinder was re-fabricated.

 The Isomet (IsoMet^®^ 4000, BUEHLER, Germany) low-speed precision cutter was used to create slices from the ceramic cylinders with the desired thicknesses and numbers, resulting in identical ceramic samples of high precision. The cutting process was performed under water coolant at a feed rate of 0.5 mm/min using a diamond disc with a diameter of 0.6 mm (Figure 1E).

 Each sample was meticulously inspected for any defects. If a defect was found, the sample was discarded and replaced with a new one. All the samples were then cleaned in an ultrasonic cleaner (Silfradent, S. Sofia, Forlì-Cesena, Italy) using 70% ethanol for 10 minutes, dried up, and subjected to polishing (Figure 1F).

###  Polishing Protocol

 All the samples were subjected to a polishing protocol using a 3-step polishing system (DIAPOL^®^ RA - Set RA 306, EVE Ernst Vetter GmbH, Germany) specifically designed for finishing and polishing lithium disilicate. The polishing procedures were conducted by one operator using a milling surveyor. ^38^

 The polishing kit comprised three steps, including three different diamond-impregnated silicon wheels: a finishing wheel (Blue), a pre-polish wheel (Grey), and a polishing wheel (white). The polishing tips were attached to a contra-angle low-speed handpiece mounted in the vertical arm of the milling surveyor to ensure consistency (Figure 1F).

 Each sample was polished on both sides at a speed of 10 000 rpm, with movement maintained in one direction. Each surface was subjected to ten strokes from each tip to achieve a highly polished surface.^39^ All samples were then cleaned in an ultrasonic cleaner using 70% ethanol for 10 minutes and dried up.

###  Colorimetry

 The samples’ primary color parameters were assessed using a spectrophotometer (RM200QC, X-Rite, Neu-Isenburg, Germany) based on the CIELab system (Figure 1G). This system uses three coordinates: L, a, and b. The L coordinate represents lightness or value, while a and b correspond to the red-green and yellow-blue axes, respectively (where positive ‘a’ values denote red, negative ‘a’ values denote green, positive ‘b’ values denote yellow, and negative ‘b’ values denote blue.^5^

 The color coordinates of the CIE Lab system (L, a, and b) were measured for each specimen three times on one side. The measurements were taken from the center of each specimen against a white background (L* = 88.81, a* = -4.98, b* = 6.09) and a black background (L* = 7.61, a* = 0.45, b* = 2.42), in accordance with the CIE standard illuminant D65. The specimens were centered in the measuring port and positioned consistently for both backgrounds. The three measurements for each color coordinate were averaged to produce a single value for each specimen on both backgrounds.

 The translucency parameter TP was calculated using the following formula^25^:

 TP = [(L*_B_ − L*_W_)^2^ + (a*_B_ − a*_W_)^2^ + (b*_B_ − b*_W_)^2^]^1/2^

 where “B” is the black background while “W” is the white background. As the TP value increases, it indicates higher translucency.

 The following formula was used to calculate the color difference according to CIEDE2000: ^18^

 ΔE_00_ = [(ΔL’/K_L_S_L_)^2^ + (ΔC’/K_C_S_C_)^2^ + (ΔH’/K_H_S_H_)^2^ + ΔR]^1/2^

 where ΔL*, ΔC*, and ΔH* represent the differences in lightness, chroma, and hue, respectively, as defined by the CIELab color space, measured between a standard and a sample in a pair. ΔR is an interaction term that accounts for the interplay between chroma and hue differences. S_L_, S_C_, and S_H_ are the weighting functions for the lightness, chroma, and hue components, respectively, and their values vary depending on the position of the sample pair within the CIELab color space. The K_L_, K_C_, and K_H_ parameters are adjustable factors for the lightness, chroma, and hue components, respectively, to accommodate various viewing conditions such as textures, backgrounds, and separations. Readings were performed by one specialist operator.

###  Accelerated artificial aging

 All the samples underwent AAA testing in the Accelerated Weathering Q-lab Ultraviolet Tester (QUV) (QUV, Q-Lab, US) (Figure 1H), following ASTMG154 06 standards. They were exposed to repeated cycles of UV fluorescent lamp irradiation at a wavelength of 340 nm and light intensity of 0.89 W/mm^2^ at 60 °C for 8 hours, followed by 4 hours at 100% humidity and away from light at 50 °C. This cycle was repeated for 384 hours, equivalent to 1 year of clinical service. ^20^ Subsequently, the samples were transferred to the spectrophotometer, and the CIELab parameters were measured again (Figure 1I).

###  Statistical analysis

 Data management and statistical analysis were performed using SPSS 20. Numerical data were summarized using mean and standard deviation. Data were analyzed for normality by checking the data distribution using Kolmogorov-Smirnov and Shapiro-Wilk tests. Based on the normal distribution of data, groups were compared using the independent t-test. One-way ANOVA was used to compare translucency across different thicknesses within the same group. Paired t-test was used for intra-group comparisons of translucency before and after aging. All *P* values were two-sided. *P* ≤ 0.05 was considered significant.

## Results

**Figure 2:**
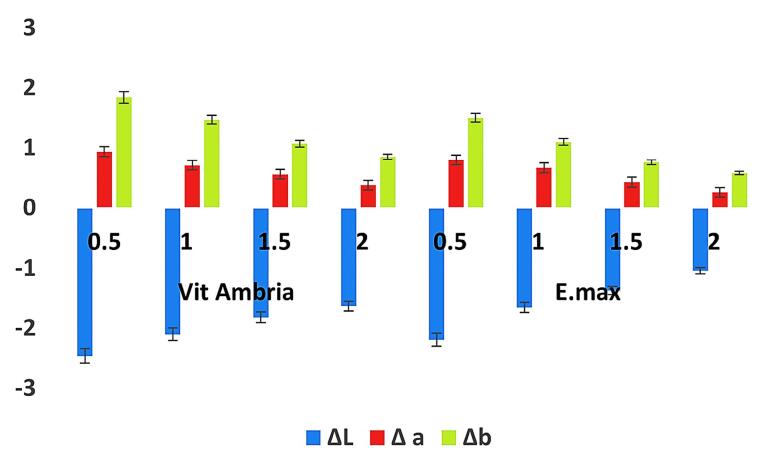
Bar chart showing mean and SD values for ΔL, Δa, Δb of various materials

**Figure 3:**
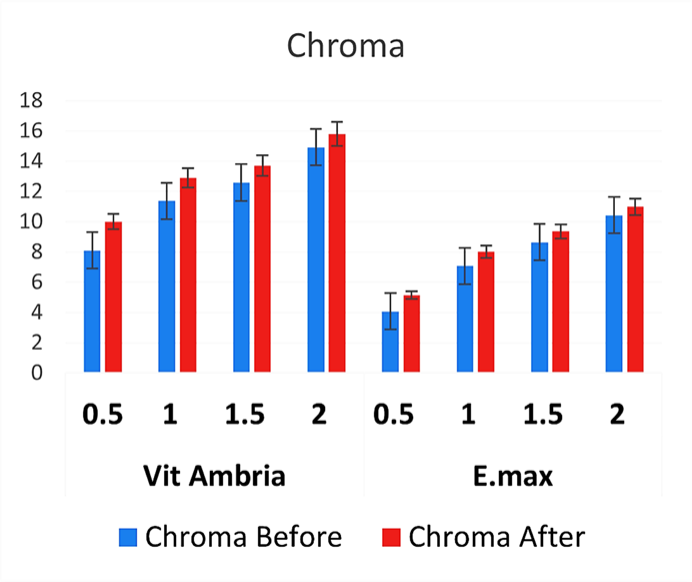
Bar chart representing mean and SD values for the chroma of the different materials

Figure 1 presents the raw data, including ΔL, Δa, and Δb calculations for LD and ZLD at four different thicknesses (0.5, 1.0, 1.5, and 2.0 mm) before and after aging. Figures 2 and 3 present hue and chroma values before and after aging.

###  Translucency

**Figure 4:**
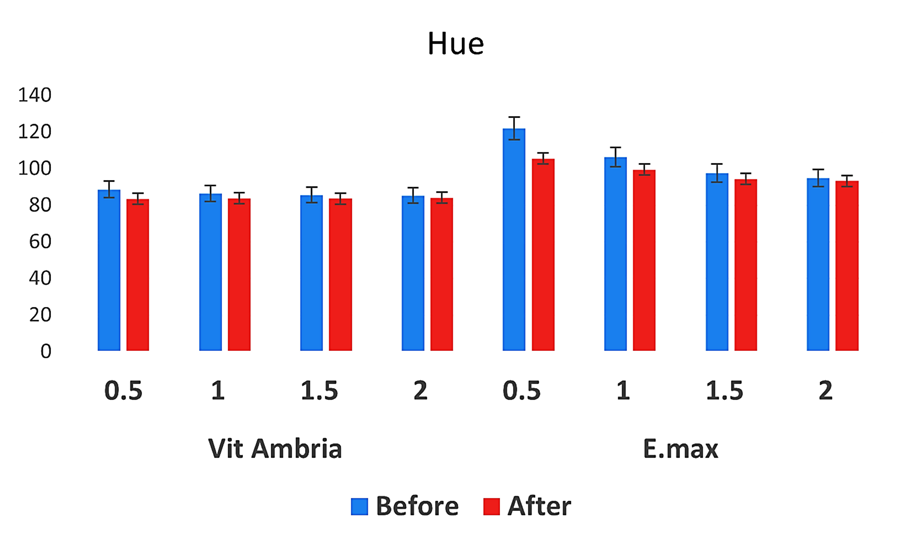
Bar chart representing mean and SD values for the hue of the different materials

Table 2 and Figure 4 summarize the results. Before and after aging, Vita Ambria exhibited significantly higher values (*P* = 0.000) at all thicknesses compared to the IPS e.max press.

**Table 2:** Descriptive statistics of translucency before and after aging

**Thickness**	**Before aging**					**After Aging**					***P *** **value of difference between before and after**
	**Groups**				***P *** **value** **between groups**	**Groups**				***P *** **value** **between groups**	
	**Vita Ambria**		**e.max**			**Vita Ambria**		**e.max**			
	**Mean**	**SD**	**Mean**	**SD**		**Mean**	**SD**	**Mean**	**SD**		
0.5 mm	16.05^a^	0.17	14.53 ^a^	0.13	0.000*	14.91^a^	0.15	13.24 ^a^	0.16	0.000*	0.000*
1 mm	11.49^b^	0.13	10.49 ^b^	0.14	0.000*	10.64^b^	0.17	9.61 ^b^	0.15	0.000*	0.000*
1.5 mm	8.76^c^	0.11	8.23 ^c^	0.11	0.000*	8.38^c^	0.12	7.73 ^c^	0.10	0.000*	0.000*
2 mm	6.84^d^	0.10	6.05 ^d^	0.08	0.000*	6.38 ^d^	0.11	5.79 ^d^	0.07	0.000*	0.000*
*P* value (thicknesses within same group)	0.000*		0.000*				0.000*	0.000*			

Significance level *P* ≤ 0.05, *Significant. Post hoc test: Within the same column, means with small superscript letters are significantly different. Within the same row, means with different capital superscript letters are significantly different.

 Regarding the effect of thickness on the translucency for both materials, post hoc tests revealed a statistically significant difference between each two thicknesses within the same material (*P* = 0.000). The recorded values showed a gradual statistically significant decrease with increasing thickness, with the highest value recorded at 0.5 mm and the lowest at 2 mm.

**Figure 5:**
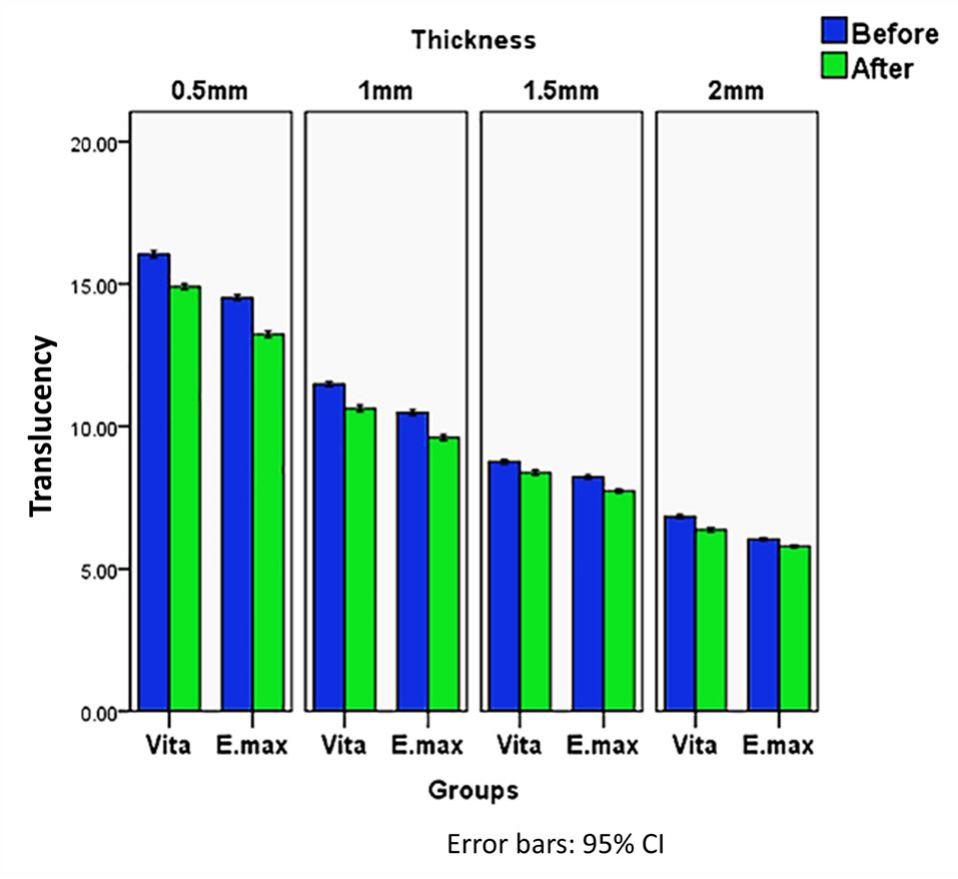
Bar chart illustrating the mean value of translucency before and after aging at different thicknesses in Vita Ambria and E-max groups

 Concerning accelerated artificial aging, the translucency was significantly reduced at all different thicknesses (Figure 5). The recorded translucency values after aging were significantly lower than those before aging across all thicknesses (*P* = 0.000).

###  Color difference ∆E2000

**Figure 6:**
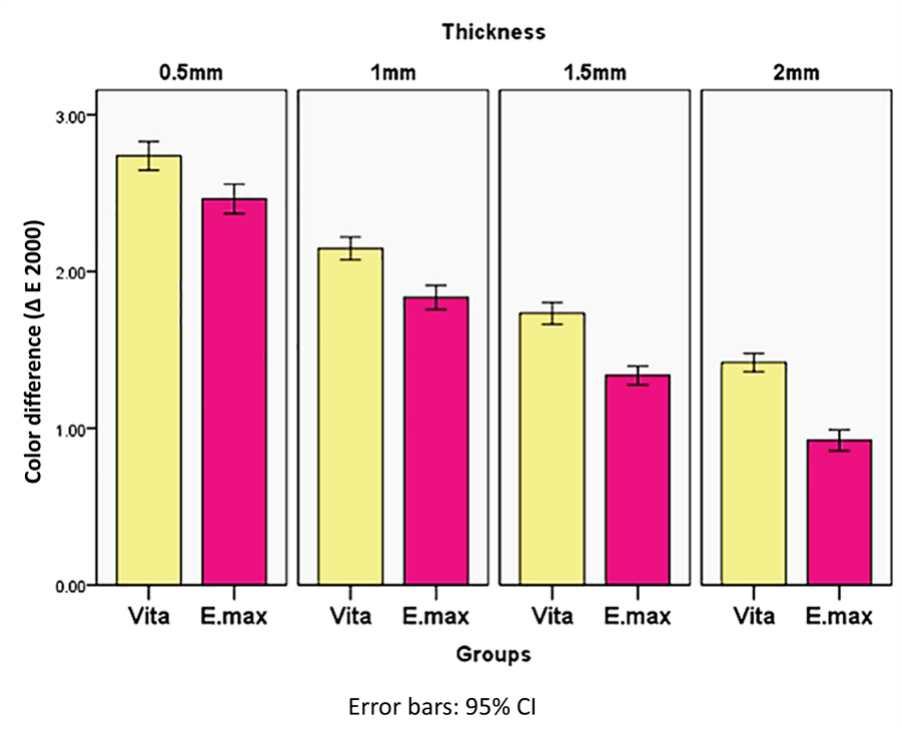
Bar chart illustrating the mean value of the color difference (∆E2000) at different thicknesses in Vita Ambria and E-max groups

Table 3 and Figure 6 summarize the results. Vita Ambria exhibited significantly higher values in each thickness compared to IPS e.max (*P* = 0.000).

**Table 3:** Descriptive statistics of color difference ∆E 2000, of the tested material

**Thickness**	**Groups **				***P*** ** value between groups**
	**Vita Ambria**		**e.max**		
	**Mean**	**SD**	**Mean**	**SD**	
0.5 mm	2.74^a^	0.13	2.46^p^	0.13	0.000*
1 mm	2.15^b^	0.10	1.84^q^	0.11	0.000*
1.5 mm	1.73^c^	0.10	1.34^r^	0.08	0.000*
2 mm	1.42^d^	0.08	0.92^s^	0.09	0.000*
*P* value (thicknesses within the same group)	0.000*		0.000*		

Significance level *P* ≤ 0.05, *Significant. Post hoc test: Means with different small superscript letter are significantly different.

 Within the same material, the recorded values showed a gradual statistically significant decrease with increasing thickness (*P* = 0.000), with the highest value recorded at 0.5 mm and the lowest at 2 mm.

## Discussion

 ZLD has emerged as a promising dental material for esthetic restorations in recent years. Its unique strength, translucency, and color stability make it an attractive choice for prosthodontic applications.^8,10,24^ However, the long-term performance of ZLD under weathering accelerated artificial aging conditions remains an area of interest and investigation. This study explored the effect of such aging on color stability and translucency, particularly concerning varying material thicknesses. By understanding these effects, clinicians can make informed decisions regarding the clinical use of ZLD restorations.

 The decision to use ZLD as the research material was based on its recent introduction to the market and the claim that it has comparable esthetics and superior strength compared to existing lithium disilicate materials, despite limited scientific evidence.^29^ In contrast, IPS e.max press was selected as the reference material due to its matching chemical composition to the test material. Additionally, it is widely recognized as the material of choice for esthetic restorations.^2,11,16^

 Lithium disilicate-based ceramics have a wide range of applications, such as veneers,^8^ crowns, onlays, ^10^ and endocrowns, ^9^ each requiring different thicknesses.^3,5,12,13^ Therefore, the use of various thicknesses in this study was intended to cover this range of applications, providing clinicians with a clear understanding of the optical properties of these materials in their different forms.

 Among the accelerated artificial aging methods, UV weathering is more suitable for colorimetric tests on dental glass ceramic materials. UV weathering can help evaluate how the material’s color may change over time due to exposure to environmental factors, including UV radiation.^15,35,36^

 UV radiation in the form of sunlight is a common environmental factor that dental restorations are exposed to in everyday use. Thus, UV weathering is more clinically relevant to colorimetric investigations than other aging methods that may not mimic real-world conditions as accurately.^15,36^

 The null hypothesis was rejected in the present study, suggesting no significant difference in the mean translucent parameter and the color difference between Vita Ambria and IPS e.max Press glass ceramics after UV accelerated aging.

 Vita Ambria demonstrated greater translucency than IPS e.max Press before and after accelerated aging. Vita Ambria is a ZLD, where zirconia acts as a nucleating agent during the pressing process.^10,28^

 Increasing zirconia content from 0 to 10% enhanced the nucleation rate, forming random, interlocking, and eventually spherical crystal structures. Higher nucleation rates lead to higher nucleation site density, resulting in smaller grain sizes and limited individual crystal growth. These smaller grains affect the refractive index, allowing more light to pass through, which increases the material’s translucency.^28^

 Translucency increases when the thickness decreases, which is true for all translucent materials because as thickness decreases, the amount of light passing through the material increases, making it appear more translucent.^21,26,40^ This finding is consistent with the results of the present study.

 Both materials exhibit a gradual and significant decrease in translucency as thickness increases after accelerated aging. The weathering of 384 hours is equivalent to one year of clinical service. ^20^ UV light and fluctuations in heat and humidity can lead to changes on the material’s surface, resulting in increased surface roughness, which subsequently causes more light scattering and reduces overall translucency.This observation applies to both materials, as they belong to the same category (glass ceramics) and share the same composition.

 For both Vita Ambria and IPS e.max Press, there was a significant color difference between the two thicknesses before and after accelerated aging. The observed color differences (negative ΔL, positive Δa, and positive Δb) after aging indicate that the material has become darker, more reddish, and more yellowish. Additionally, the material became more saturated (increased chroma) yet has a different hue, which typically suggests rotation of the hue angle in the color space. Similar results were reported by Choi et al.^36^ This combination of changes is typical of many glass ceramic materials exposed to environmental aging factors like UV light, humidity, temperature fluctuation, and oxygen, which alter the material’s appearance over time.^14,37^ Also, abrasion and surface wear can alter the reflectance properties, leading to a darker appearance and changes in hue.^28^

 As the thickness decreases, the changes in ΔL, Δa, and Δb values become more apparent, indicating greater color shifts. This suggests that aging makes thinner samples more susceptible to visible color changes. Hue and chroma values also showed notable changes, further highlighting the impact of thickness on color stability.

 Thicker material tends to absorb more light and scatter it internally, resulting in a more consistent appearance. In contrast, thinner material allows more light to pass through, making the color appear lighter and more transparent. This transparency can make the material’s color more susceptible to the influence of the surrounding environment.^21,22,41^

 The color difference was more evident in Vita Ambria than in IPS e.max Press samples, indicating higher color stability of IPS e.max Press. Vita Ambria experienced more color changes, possibly due to the presence of zirconia particles.^29^ While zirconia in glass ceramic has benefits, it also has drawbacks. Adding zirconia increases porosity, which rises with higher zirconia content due to increased viscosity. This porosity can allow stains, colorants, and fluids to penetrate, leading to instability of the material and color change over time.^28,30^

 In the current study, according to ∆E2000 acceptability and perceptibility limits (0.8 and 1.8, respectively),^18^ the color was deemed clinically acceptable when the thickness was more than 1 mm. However, when the thickness was ≤ 1, the color change was considered unacceptable. This highlights the significant effect of the thickness of the lithium disilicate-based ceramics on their final esthetics.

 Phark and Duarte^30^ found that artificial aging influences the translucency and opalescence of lithium disilicate but is less significant compared to zirconia-reinforced lithium silicate glass ceramic or feldspathic glass ceramic. This finding was supported by a systematic review by Potdukhe et al,^41^ in which they investigated the effect of artificial aging on the translucency of zirconia-reinforced lithium silicate and lithium disilicate ceramics, consistent with the current study.

 Rizk et al^14^ found that Vita Ambria was more translucent than IPS e.max Press after thermocycling, which affected the translucency and color stability of both materials, consistent with the current study.

 The limitations of this in vitro study include that it did not fully replicate the complexity of the oral environment. Additionally, the study only included two materials. Moreover, the investigation did not consider the influence of cements on the translucency of the tested materials. Therefore, further studies are needed to explore the impact of cements on the translucency of ceramic materials, broaden the scope to include more ceramics, and observe color changes in clinical studies.

## Conclusion

 Within the limitations of this study, the following conclusions can be drawn:

1. Vita Ambria showed greater translucency than IPS e.max press across all thicknesses, indicating its suitability for esthetic dental restorations.
2. Both materials experienced a significant decrease in translucency after accelerated artificial aging, suggesting that UV exposure and temperature fluctuations can affect the esthetic outcome and durability of dental ceramics.
3. Translucency was inversely proportional to material thickness, with thinner samples showing greater color differences and translucency, emphasizing the impact of thickness on color stability and translucency.
4. IPS e.max press was more color stable than Vita Ambria.
5. UV aging affected the color parameters of lithium disilicate-based ceramics, causing the material to become darker, more reddish, and more yellowish.

## Acknowledgments

 We extend our sincere gratitude to our mentors for their invaluable guidance and to the crown and bridge department team for their support.

## Competing Interests

 There were no conflicts of interest.

## Ethical Approval

 This study was ethically approved with code 678/1660 by the Ethics Committee at the Faculty of Dental Medicine, Al-Azhar University, Cairo, Egypt.
